# How P(H)C is your curriculum?

**DOI:** 10.4102/phcfm.v16i1.4590

**Published:** 2024-09-09

**Authors:** Bernhard M. Gaede

**Affiliations:** 1 Department of Family Medicine, School of Nursing and Public Health, University of KwaZulu-Natal, Durban, South Africa

**Keywords:** health professions education, curriculum design, primary healthcare policies, PHC, curriculum review

## Abstract

In South Africa and internationally, the alignment of health professions education programme with primary healthcare (PHC) policies is seen to promote the training of fit-for-purpose graduates who can adequately respond to the demands of patient and community needs. This article seeks to describe the development of a tool to assess the degree of PHC in an undergraduate medical curriculum. In defining what is meant by PHC, four dimensions of PHC were identified for the purpose of designing the tool, namely values underpinning PHC, principles of PHC, a generalist focus of the programme, and the level of care that the programme is delivered at. The tool also sought to assess how the content in these dimensions is covered in the curriculum and to which depth students are required to engage. The perspectives that were considered were: what content was being covered, what pedagogy was used, in which context it was being taught and how it was being assessed. For each of these aspects, the dimensions are assessed using an amended Miller’s pyramid to assess the expectations of outcomes for the curriculum. The tool is presented and the article reflects on the use of the tool in a process of assessing a medical curriculum.

## Introduction

In order to adequately respond to the changing burden of diseases and demands of the healthcare system, policies and guidelines for health professions education in South Africa and internationally increasingly require that health professions education become more aligned with the primary healthcare (PHC) approaches.^1,2^ The envisaged fit-for-purpose health professional graduates would be well prepared to be able to work at the frontline, be more responsive to patient and community needs and function optimally in the healthcare system of the country.^3^

However, while there is considerable rhetoric about aligning with PHC, what exactly is meant by it is less clear, so it is imperative to define what we mean by PHC. Any changes in the curriculum towards PHC would need to be assessed in terms of an understanding of what PHC is, and how it would be evident in the curriculum. It requires agreed-upon metrics to assist in tracking curricular design and changes.

This article seeks to describe a tool that was developed to assess to which degree PHC was represented and taught in the current undergraduate medical curriculum. For this, a working definition of how we understand PHC was developed.

## The four dimensions of primary healthcare

There is a tension around how the term PHC is being used. Drawing on the wealth of published literature, a working definition for PHC in the curriculum was developed. In some instances, ‘PHC’ relates to a philosophy or value system that underpins health systems development (such as equity and social justice),^4^ while PHC may also refer to a set of principles that describe the characteristics of a service (such as person-centredness,^5^ or accessibility, availability, acceptability^6^). Within the healthcare system, PHC often refers to a level of care where the first contact with a person happens (as opposed to secondary or tertiary care),^7^ or it may refer to an integrated ‘basket of services’ (whether comprehensive or programmatic) that are available at the first point of contact with the healthcare system.^8^

To develop a comprehensive description of PHC, the core aspects of PHC have therefore been arranged around four dimensions outlined as follows:

### Dimension 1

The value system underpinning PHC:

- Social accountability.
- Social justice and commitment to human rights, equity and humanism.
- Commitment to social action and agents for change (advocacy, agency and working in disadvantaged and marginalised communities).

### Dimension 2

Orientation-principles of PHC:

- Person-centered approaches – placing the person (not the disease or organ system) at the centre of the curriculum.
- A multicultural competency and cultural safety approach to healthcare including indigenous knowledge systems and plural healthcare systems.
- Ecological understanding of health beyond biomedical perspective – linking the individual to the family, community, population and global dynamics (biopsychosocial approach and public health perspectives).
- Focus on key social determinants of health, including lifestyle, gender-based violence, inequity, poverty and structural violence.
- Link care processes explicitly to prevention of disease and health promotion as well as to rehabilitation and palliative care, that is, primary, secondary, tertiary and quaternary prevention.
- Evidence-based approaches.
- Participatory approaches including community-oriented primary care and intersectoral community action sustainably addressing upstream factors.

### Dimension 3

Content-generalist focus versus specialist focus:

- Content covers a core curriculum of common conditions with generalist approach to management.
- Integration of approaches, tools and portfolios across disciplines, that is, not discipline-specific approaches.
- Interprofessional education and team approaches to management of patients and conditions.
- Nurturing relationships in teams, among individuals and across hierarchies.

### Dimension 4

Context-level of care:

- Community-level care and engagement with resources outside of the healthcare system.
- Primary healthcare clinics and district level of care for clinical and practical teaching and exposure.
- Inclusion of managing care across levels of care and across disciplines, including referrals, continuity of care and support for care in the community.

There are many aspects of PHC that may have not been covered in the description earlier, attesting further to the complexity of the term and the wide and varied use of it. While most of these dimensions are relatively generic, for specific disciplines, particular dimensions of PHC may be of greater importance. The tool was developed for the medical curriculum, and if a similar tool was to be developed in other disciplines, it may look different. As an example in the rehabilitation professions, a greater focus on community rehabilitation services may be a specific area to be assessed, rather than PHC clinics.

## Review of the curriculum

Besides exploring the dimensions of PHC in the curriculum (as outlined earlier), a vital part of the assessment is also to explore how the content is covered and to which depth the students are required to engage. For our review, the following perspectives were considered:

- *What* is being taught and learnt (content).
- *How* it is being *taught*, what is being learnt and what changes (pedagogy).
- *Where* it is being taught (context).
- *How* it is being *assessed* (outcomes).

For all of the dimensions, we further considered an amended Miller’s pyramid^9^ as part of the spiralled curriculum:

- Know: what is the basic science for each of the dimensions?
- Know how: what is the applied science for each of the dimensions?
- Show how: what are the skills and procedures required to implement the knowledge?
- Do: what are the outcome function as a fit-for-purpose professional?
- Being: the embodiment of professional values required as fit-for-purpose professional.

## Development of a tool and how it can be used

The dimensions and perspectives that have been described earlier have been consolidated into a matrix for the assessment of the curriculum. In the tool, the dimensions were listed in a column, and the range of extent and depth of teaching was covered at the top (see Appendix 1).

### The use of the tool

The tool was used in two distinct phases. Firstly, it was applied to the documented evidence of programme and module templates as well as the curriculum map to assess whether any of the PHC aspects outlined earlier was evident. Two separate reviewers scored the documentation on the tool and the results were compared and collated. Where there were differences, these were discussed and a consensus decision was taken.

Secondly, in a process of engaging with all of the module coordinators, the tool was completed jointly through discussion about what was actually being taught and whether aspects of PHC were included. The approach used was an appreciative inquiry approach that allowed for considerable discussion and exploration of possibilities rather than only extraction of information. The process of jointly completing the tool ensured that the shared information was captured accurately and reflected the nuances in the comments that were offered. The discussion also expanded beyond the strict confines of the focus on PHC and included very valuable information about pedagogic approaches more broadly, issues of coordination of the module and the relationship of the module to the whole programme.

The results from the two approaches were then shared with the faculty involved in the medical programme as part of the curriculum review process and it was followed with a wider curriculum renewal process.

## Reflection on the use of the tool

The tool proved to be very useful to get an overview of PHC in the curriculum. It assisted in generating discussion regarding the definition(s) of PHC and moved away from the broad, generic statements around whether PHC was addressed in the curriculum and to which degree. The much more nuanced discussions regarding PHC also provided useful perspectives on how PHC could be incorporated in many situations that are not traditionally thought of as PHC, such as discussion on referral systems or follow-up of complex patients in the community.

The approach of doing both the document review and the appreciative enquiry engagement with the faculty teaching the modules proved to be a useful start to conversations regarding the curriculum as a whole. It generated critical discussion, engagement and exploration around the current practices and future possibilities.

While some aspects of the tool allowed for relatively exact measures (especially regarding the location and context of teaching, e.g. what amount of time in the curriculum do students spend in community settings or in primary care clinics), many aspects of the findings were not as easy to measure in exact metrics. The tool also did not explore other aspects of the curriculum, such as the overall pedagogy or how the teaching of PHC related to other aspects of the module. While this was explored to some degree in the discussions that the process generated, it was more a function of the process and how the tool was used, rather than the tool itself.

## Conclusion

The initial steps of reviewing and redesigning the curriculum include a needs analysis and a more formal assessment of the current curriculum.^10^ The tool described earlier served this purpose in our context, and while it worked well to serve our needs, it was not designed to be used as a standard for the assessment of the curriculum. From our experience, it was perhaps the *process* of using the tool that was more useful and important than the tool in itself. Systematically reviewing the curriculum with a clear question in mind has been very valuable. However, while the tool was not designed to address the wider curricular issues, it ended up generating a nuanced and detailed understanding of the curriculum as a whole and generated extensive discussion. Even with limited scope of the tool, it has been a very useful exercise to engage in.

## Appendix 1

### Copy of tool

#### Check list for PHC in the curriculum

Date:

Module:

Participants:

**TABLE 1-A1:** Section 1: Coverage of PHC.

Category	Indicator	Definition/focus	Theory/content	Lectures, practicals, group work, PBL cases	Assignments, continuous assessment	End-of-module assessment
			Is it being taught?	How is it being taught?	Are there tasks on it?	How is it being assessed?
Values underpinning PHC	Social accountability					
	Social justice, human rights					
	Social action					
	Change agency					
Principles of PHC	People-centredness					
	Cultural competency					
	Ecological perspectives					
	SDH					
	Promotion prevention Curative Rehabilitation Palliation					
	Intersectoral action					
Generalist focus	Core curriculum					
	Integration of tools					
	IPE					
	Teams and relationships					
Level of care	Community level					
	PHC/district hospital					
	Referral system and continuity of care					

**TABLE 2-A1:** Section 2: Depth of PHC.

Category	Indicator	Know	Know how to	Show how to	Do
Values underpinning PHC	Social accountability				
	Social justice, human rights				
	Social action				
	Change agency				
Principles of PHC	People-centredness				
	Cultural competency				
	Ecological perspectives				
	SHD				
	Promotion, prevention Curative Rehabilitation Palliation				
	Intersectoral action				
Generalist focus	Core curriculum				
	Integration of tools				
	IPE				
	Teams and relationships				
Level of care	Community level				
	PHC/district hospital				
	Referral system and continuity of care				

*Source*: Based on Al-Eraky M, Marei H. A fresh look at Miller’s pyramid: Assessment at the ‘Is’ and ‘Do’ levels. Med Educ. 2016;50:1253–1257.
